# Correction: The use of paracetamol during pregnancy: A qualitative study and possible strategies for a clinical trial

**DOI:** 10.1371/journal.pone.0318023

**Published:** 2025-01-17

**Authors:** Cathrine Vedel, Ditte Staub Jørgensen, David Møbjerg Kristensen, Olav Bjørn Petersen, Gorm Greisen

The Funding statement for this paper is incorrect. The correct Funding statement is as follows: O.B.P. holds a professorship funded by Novo Nordisk Foundation, grant NNFSA170030576.
